# Practical Pathology of the Mucous Membrane of the Nose, Throat, and Ears of Youths

**Published:** 1896-07

**Authors:** Thomas F. Rumbold

**Affiliations:** St. Louis, Mo.


### PRACTICAL PATHOLOGY OF THE MUCOUS MEMBRANE OF THE NOSE, THROAT, AND EARS OF YOUTHS.

NUMBER III.

BY THOS. F. RUMBOLD, M.D.,

ST. LOUIS, MO.

*The Gradual Increase of the Diseased Condition.* If a pieee of injected turbinate process from a subject in this grade is placed under a microscope, the abnormal condition of the mucous glands, the increased diameter of the blood-vessels, the irregularity of their course and diameter, positively preclude the supposition that the inflammation can be only a few years old. The whole appearance asserts that the disease is nearly equal in age to that of the subject. Not only this, but the extension of the disease to other parts contiguous or connected by nervous or vascular structure becomes more and more manifest, extension that is physically impossible in a shorter length of time.

*The Nasal Passages in Youths*—aged from ten to twenty years—exhibit proportionately greater evidence of inflammation than those of the younger grades; some of the surface blood-vessels are enlarged so as to be plainly seen with the unaided eye, the largest being from ten to forty times their normal diameter. There is a general increase in color due to the increase of the age of the inflammation. The secretion has more pus in it, and is thicker on account of evaporation caused by increased heat. Frequently it emits a fetid odor, due entirely to its retention in the passages.

If a piece of mucous membrane of the locality where this fetid secretion adheres is examined under the microscope, many of the largest mucous glands will be seen to be very much enlarged and distorted, as well as abnormally full of unexpelled fetid secretion. From many of these enlarged glands shred-like projections of the tenacious fetid secretion hang outward. If a solution of permanganate of potassa is applied with a brush, these shred-like projections are colored, the color being exactly the same as seen in life, when this solution is employed in cleansing a nasal passage; the more diseased the membrane the higher the coloring, showing that it is the secretion principally that is colored. Under the microscope it is seen that no part of the mucous membrane is so much colored as is the part where the fetid secretion adheres. This proves that while the permanganate may deodorize the fetid secretion that is on the surface, it does not remove it, for if it did remove it the part would not be so highly colored. This microscopic demonstration also shows why the peroxide of hydrogen cannot cleanse a nasal passage thus affected; it may cleanse all the secretion outside of the glands, but cannot get inside of them, their mouths being too small. The patient cannot tolerate the quantity that would be required to deodorize the secretion inside of the glands.

Many have insisted that the fetor comes from the mucous membrane itself, because the odor persists after the passages have been thoroughly washed out with an antiseptic water. The microscope proves this to be a mistake; the fetor comes from the unremoved secretion. Cleansed mucous membrane emits no odor, certainly not the one under consideration. Even if a brush, or a sponge, or a cotton applicator *could* reach all the surface covered by this secretion, none of them can be relied upon to cleanse, such is the tenacity of the secretion. If this is the case, should we expect a watery solution, warm or cold, to remove it, especially as we know that water will not dissolve it? The effect of warm water or other astringents—for water manifestly acts as an astringent—prevents the normal functions of the membrane, namely, the formation of mucus. Besides this, water is absorbed by endosmosis and increases the disability of the mucous membrane; the proof of which is that the patient takes cold more frequently and severely after its use.

*The mucous membrane forming this kind of secretion has not been made clean by any kind of human effort that has yet been tried.* Since 1875 I have not used water in the nasal passages; from 1862 to 1874, inclusive, I unsuccessfully employed water—many times every day, in all bearable temperatures, in combination with every material, amounting to several hundred kinds, that I was sure would not be a positive injury. After the use of the watery solution, I faithfully and patiently tried, sometimes by the hour, to complete the cleansing with a small brush, with a soft sponge, and with absorbent cotton. I used the faradic and galvanic currents in combination with all these, without cleansing the nasal passages *in one case.*

This kind of secretion clings to the outside surface as well to the inside surface of the mucous glands *purely* because there is no normal mucus formed to wash it off, and until mucus that is nearly normal, at least, washes this secretion away, it will remain where it is seen to adhere. This is a very valuable pointer from pathology; the wise will heed it.

*The Turbinate Processes.* The enlargement of these projections keeps pace with the increase in the size of the nasal passages. Frequently one of the inferior turbinates is as large as the end of the patient's little finger. The blood-vessels, which make up the principal bulk of the inferior turbinates, are proportionately increased in size, and are a little more irregular in their course than are those in the younger grades. If colds have been frequent the enlargement may be so great as to completely occlude the passage. It is seldom that this occlusion continues throughout the day; when it does so, then tonsil-growths will be seen, and sore throat will be frequent each fall and spring. Of course this condition soon makes the sufferer a mouth-breather, but mouth-breathing is not commonly started in this grade. If the sufferer does become a mouth-breather at this time of life, this disability, while it is not complained of by those in the younger grades, is now so decidedly uncomfortable and is so noticeable to others that a physician is consulted at once. The patient may keep his mouth so slightly open as scarcely to be noticed, but the complaint will soon so increase in severity as to compel him to seek relief. If he does not get relief, he may have an attack of pruritic rhinitis (hay-fever) or asthma, or other lung trouble.

*Nose-bleed* is a symptom of acute inflammation; that is, a sudden engorgement of the blood-vessels, one that bursts the vessels and allows the blood to flow. While this is not of very frequent occurrence, yet it is more common in this grade than in the older or younger grades. The reason why it is not more common is because the nasal inflammation has now become quite chronic; the thickening of the walls of the blood-vessels has kept pace with the increased size of the vessels. When hemorrhage does take place, it indicates that the patient's system—that is, his stomach, bowels, and kidneys—has been out of order, or that there is an excess of fluid in the blood, and at the same time a severe cold in the head has been taken so as to suddenly increase the inflammation. In this case the pressure of blood in the blood-vessels has been greater than the thin walls of the blood-vessels could withstand; consequently rupture followed, frequently to the great relief of the sufferer, especially if the hemorrhage came from the blood-vessels having their origin from cranial vessels.

*Tumors*, such as gelatinous and fibroid—really one kind of tumor but of different aged growths—are common in this grade. Most frequently they are attached to the most dependent portion of the turbinates, showing plainly that their cause is due to the irritating presence of acrid nasal secretion. All tumors or growths in the nose, throat, and ears have the same cause, namely, irritating secretion from an inflamed mucous membrane. The proof of the correctness of this assertion is that the treatment of the inflamed parts will cause the disappearance of lately formed growths. I have seen in almost hundreds of instances small gelatinous growths in the nose, throat, and ears disappear while preparing the parts for an operation; that is, while reducing the inflammation by treatment so as to prevent excessive hemorrhage and accelerate the healing process after the operation. I have a patient who visits me a few times each fall and spring upon whom I discovered a small tumor on the left vocal cord in 1884. After treating him for a little over one week the tumor disappeared. After an absence of two years he returned—so had the tumor, on the same spot. A

ten-days' treatment caused it to disappear again. In about fifteen months it reappeared, to go away with a week's treatment. Its recurrence was the only fact that caused alarm.

*Enlargements and Growths* of various kinds usually commence in this grade; sometimes they are so small that it requires the eye of an expert to detect them. Every swelling or enlargement, if maintained, is *sure* to become a growth, and one growth is *sure* to increase congestion, and this has a tendency to produce other growths. This is invariable, and the sooner it is known the better for all concerned, and the number concerned is legion. These serious results are solely due to a mechanical cause and one that is not at all difficult to understand when plainly stated; it is as follows:

*The Mechanism of Growths.* Every swelling in the nasal passage takes away air-space, thus preventing normal respiration. As every inspiration *must* draw in a certain amount of air into the lungs (and this normal requirement cannot be lessened even to the extent of five cubic inches), the curtailment of air-space *must* increase the rapidity of the stream of air so as to maintain the normal quantity of air in the lungs.

During inspiration through the normal nasal passages there is a slight rarefaction of air in all parts of the respiratory tract, and during expiration there is a slight condensation of air in the same passages. This is the normal condition, but a deviation from this, even to a very slight degree, *must* result in producing a more or less abnormal condition not only in the nasal passages but in various contiguous organs and those connected by vascular and nervous structures. It is evident that even a very slight swelling in one nasal passage *must* lessen the air space, and this lessening *must* cause greater rarefaction of air than normal, and this air-exhaustion, acting as an air-pump on the blood in the blood-vessels *must* increase the congestion, and this increased blood-supply *must* increase the growth, which means still greater air-exhaustion followed by increased diseased action, until the life of the patient is taken, not by this growth alone, but by consequential diseases that frequently follow this diseased condition.

The contrast between the excess of air-exhaustion and the excess of air-compression becomes greater with the increase of the growth in the nasal passages. These abnormal conditions cannot be continuously maintained without positive injury to every organ connected with the organs of respiration: The head and brain through the nervous connections and through the cerebro-nasal circulation, the eyes through ophthalmo-nasal circulation, the ears through the disturbance of the normal density of the air as it passes up into the Eustachian tube, the larynx through its continuity of structure and by vascular and nervous connections, and the lungs by the abnormal extremes of air density.

*Cartilaginous and Osseous Over-Growths of the Septum Nasi.* While these over-growths are not very marked in this grade they always commence in these ages. The cause of their appearance is the same as that of other growths, namely, continued excessive blood-supply due to maintained congestion of the perichondrium and periosteum. The growth usually appears on one side only, at first more frequently on the left.

*The Site* of the appearance of the growth is important, as it indicates the source of the irritation that starts and maintains it. I have yet to see a growth start on the septum where there was an absence of accumulated irritating secretion. If the growth is well inside the nasal passage, it will be found just opposite one of the turbinates.

No growth, such as the kind under consideration, is seen on the posterior fourth of the septum, for the reason that none of the turbinates touch this portion. There is an over-growth of cartilaginous structure seen on the anterior portion of the septum, almost at its anterior extremity, that at no time is in contact with an acrid-secretion-holding surface, but the history of each of such cases invariably indicates that for a long time an acrid secretion has been continuously flowing over that part of the septum, thus maintaining the irritation that is observed to always precede and maintain these growths.

The most usual site for these over-growths is opposite the left inferior turbinate. If this turbinate is large enough to touch the septum so as to maintain acrid secretion on it, a growth will be seen here; if the turbinate does not at any time, day or night, touch it, no growth will be seen. When the septal growth is small and the turbinate is seen to be not within a quarter of an inch of the septum, the only evidence of the contact during the previous night is small threads of mucus stretched across the passage from one side to the other. These slender strings are positive proofs that the two surfaces were in contact, and the position of the septal growth and the enlarged turbinate strongly indicate the relation of cause and effect. There is another proof that the septal growth is due to irritating secretion held to its surface. It has just been stated how the inferior turbinate assists to form a growth. Instances are quite common where the enlarged superior or middle turbinate of the opposite nasal passage has assisted to form a septal growth on this side. These higher growths are smaller because of the less amount of secretion held by these turbinates, and because of the secretion being less acrid.

If the inflammation now affects both sides of the lower portion of the septum, the increase of the cartilaginous or osseous structure, or both, will compel the newly grown substance to go toward the side of the greater disease, which is usually the side first affected. As this enlargement takes place, room for the increased area of the structure must be found. As it cannot force the bridge of the nose upward, or the hard palate downward, it must become deflected to one side, and that side is toward the side with the greatest inflammation.

It is seen that the septum may be deflected toward the left side in its lower portion, and toward the right side in its upper portion. I have seen these conditions certainly from one to three times every month during the last thirty-five years.

*The Cause of the Deflection of the Septum.* Mr. Collier, in the *Medical Press and Circular*, November, 1895, very strongly insists that the deflection of both cartilaginous and bony septum nasi is due to the partial occlusion of that side of the nasal passage that holds the growth. The breath during inspiration in a partially occluded passage causes a partial exhaustion of the air in the cavity, the tendency of which is to pull the septum over to that side, just as the air-pump would do. While I believe that he is correct in this, I think that there is *another agency* also at work, and that is

—as I have stated above—the tissue-increasing inflammatory process itself. Say that a small growth starts in the left nasal passage, the septum on the right side is still straight. As the growth increases, a slight concavity—not much more than one tenth the size of the growth—is observed on the right side of the septum. As the growth increases so does the concavity, but, be it remembered, not nearly to the same degree as does the growth. There is no doubt that the whole substance of the septum becomes involved in the growth, but the protrusion of the growth-side is always far greater than the concavity of the other side. It is seen that the tendency of the growth is to go toward the most diseased side. The instances in which the lower portion of the septum is greatly deflected to the left, and the upper portion nearly as greatly deflected toward the right, prove that I am right as to one cause of the deflection.

It is nevertheless evident that the growth causes air-exhaustion, and this air-exhaustion draws the growth toward the rarefied side, thus assisting in causing a deflection of the septum nasi. And to Mr. Collier we are indebted for this idea. This view of the matter emphasized the importance of the removal of all growths and deflections.

*Diseased Accessory Cavities.* Of the last hundred cases in this grade that have been under my care, about twenty had undoubted symptoms of disease of one of the accessory cavities. Of late years I am seeing a greater percentage than is here indicated; not that these kinds of cases are increasing, but that my ability to discern them is increasing. Many of the cases that were designated, fifteen and even ten years ago, as atrophic rhinitis were really a diseased condition of one or more of these cavities. Disease of the sphenoid and ethmoid cells, in connection with the appearance of adenoid growths, has been mentioned. As age advances the formation of the superior and posterior portions of the nasal cavities changes, so that the secretion does not as readily flow down to the region of the adenoids, more of it remaining on the superior turbinate process because it is partially inspissated on account of increased heat. If the accumulation is large it may affect the breath.

*Locating the Disease by a Cotton Pledget.* There is but one method of proving positively whether or not the accumulated secretion is the overflow of the sphenoid and ethmoid cells or is thrown off by the mucous membrane covering the superior turbinate, and that is to place a small pledget of cotton on this turbinate process, and allow it to remain there for twenty-four hours. If the process secreted the secretion the cotton will appear clean and the secretion will be under it; if the secretion came from the sphenoid or posterior ethmoid cells, then it will be on the top of the cotton.

If the anterior ethmoid cells are diseased, the outflow will accumulate, provided the secretion is thick, on the middle turbinate. The application of cotton, as mentioned above, will prove whether the secretion comes from the cells or is formed by the mucous membrane of the process.

If the frontal sinuses or the antrum of Highmore is diseased, the secretion from these cavities will lodge on the inferior turbinate process. The cotton, if applied on this process, will prove the origin of the secretion, as it did in the case of the other two turbinates.

*Atrophic Rhinitis.* The most prominent objective symptom of this condition is an atrophy of the mucous membrane of one or more of the turbinate processes, most generally the inferior. The membrane has passed through the stage of hyperplasia, by many called hypertrophic rhinitis, and has now become reduced in thickness because of the atrophy of all its parts, the blood-vessels, the glands, and all the other structures that form it.

There is no doubt but that there are cases that are affected with atrophic rhinitis and ethmoiditis at the same time; but a case in this grade affected with atrophic rhinitis alone almost never consults the physician for this complaint, for it will cause no more trouble than an atrophic tonsil-growth or an atrophic adenoid-growth in the pharyngo-nasal cavity.

The application of cotton to the region of the location of the secretion will positively indicate the location of the formation of the secretion. It must be kept in mind that an atrophic mucous membrane secretes but very little mucus, and but little or no muco purulent secretion. If the cotton is applied to a suspected case of atrophy, the location of the secretion (on the top of it or under it) will indicate the kind of case. If on the top, then it is one of ethmoiditis; if on the mucous membrane, so the cotton is clean, then it is a case of atrophic rhinitis. If the secretion is on both the top of the cotton and on the mucous membrane, then both diseases are present.

*Erectile Tissue.* This is usually seen on the anterior extremities of the inferior turbinate and on both sides of the posterior portion of the septum nasi. This growth is due to an enlargement of the blood-vessels of the part. These blood-vessels are permanently abnormal, and must be treated mechanically.

*Adenoid Growths* are not seen, as a general thing, in this grade; but if a continuous stream of acrid secretion from the sphenoid and posterior ethmoid cells is forced to flow down the pharyngo-nasal cavity because of an obstruction by a growth in one or both nasal passages, this stream, acting as it did in the infant, will continue to maintain the growth to the age of childhood and even until the sufferer reaches the adult age, as I have seen on a number of occasions.

*Elongation and Enlargement of the Uvula.* This is always due to excessive and long-continued inflammation. Very frequently the enlargement and elongation commence immediately after a prolonged attack of edema.

*Tonsil Growths or Tonsilloids* are seen in about 80 per cent. of this grade. It is not generally known that these growths are the most vacillating as to their size of any abnormal growth of the body excepting the inferior turbinate. If the patient is under treatment and in good health, the growth will shrink fully one-quarter of its size in two or three days, to again increase in size on taking cold. I have had a few cases where these growths entirely disappeared inside of three months upon the institution of nasal treatment alone. This course is not to be recommended, for the reason that the tonsil-growths are quite likely to reappear, but I give it to show the relation of the growth to the nasal disease. Sometimes there are accretions of chalky material in the body of the growth, sometimes fetid balls are formed and thrown out during a coughing spell. Frequently these balls give the breath a very disagreeable odor. Abscesses over these growths are very common.

*Follicular Pharyngitis* begins now to appear. These are sprouts or growths from the glandular structure of the wall of the pharynx, just as an adenoid is a growth from the glands in the pharyngo-nasal cavity, the tonsil-growths from the tonsils, and lingual-growths upon the base of the tongue. While they give rise to a tough, stringy secretion, they usually occasion but little disturbance. If the secretion from them is so much as to cause the patient to continually clear his throat, they ought to be removed; but it must be kept in mind, however, that the occasion for clearing the throat is far more frequently—ten to one—caused by secretion formed in the posterior nasal passages and the upper pharyngo-nasal cavity than from these follicles.

*The Curvature of the Epiglottis* of an infant is equal to a segment of a circle of about one-quarter of an inch in diameter, that of a child about one-half an inch, that of a youth about five-eighths of an inch, while that of an adult is that of one inch.

If an infant has a very severe inflammation of the throat, so as to involve the epiglottis, this organ will be so affected that the curvature that it then has will be maintained the remainder of its life; that is, the curvature of the epiglottis will not increase with the growth of the larynx but continue the curvature that existed at the time of the disease of the throat. The growth of the epiglottis will be in the extension of the arms that extend from the curved portion, so that if the free upper edge of the organ was straightened out it would measure as much as that of a normal epiglottis. The abnormality is in the shape of its free upper border.

If an epiglottis of this kind is examined with the microscope it will be seen that the perichondrium has become "thickened and more vascular, and the blood-vessels more numerous and larger in caliber. Most of the glands on the posterior surface are either in a proliferous or atrophied condition. The smaller the curve of the epiglottis, the younger was the patient when the inflammation occurred."*

If on examination of an adult patient the curvature of the epi-

*Treatise on "Nose, Throat, and Ears," Rumbold, page 181. 1888.

glottis is equal to a segment of a circle or a quarter of an inch in diameter, then the inflammation occurred in infancy; if the curvature is equal to a segment of five-eighths of an inch in diameter, then the disease occurred in childhood, and so on.

These curvatures are permanent, and have quite a modifying effect on the voice.

*Disability of the Voice.*—Almost every disability of the voice is occasioned by inflammation of the pharyngo-nasal cavity. The presence of the tonsil-growths frequently has a bad effect on the voice in singing. It is not an uncommon occurrence for patients to gain from two to four notes in their singing register after treatment and operations removing the tonsil-growths.

There is an abnormal spot near the mouth of the Eustachian tube, that if touched with pharyngeal mirror, or by a medicament forcibly applied, will instantly affect the voice, and sometimes take it away altogether for a few seconds or minutes. Sometimes in the same cases, a gentle pull of the pinna of the ear will produce the same vocal disability. Very guarded promises should be given in such cases, as they frequently are remarkably slow in recovering the normal tone and use of the voice, especially in singing.

*The Ears.*—The inflammation of these organs is usually a continuation of that of childhood, and that back to infancy. The Eustachian tube is the part first affected. This canal may be abnormally closed—by far the most usual condition—or abnormally open, a condition that is difficult to diagnose, one to which I have given a great deal of study. It was while studying this diseased condition that I discovered the functions of the Eustachian tube.

The closure of the Eustachian tube may be acute or chronic. In this grade it is seldom acute. When it is acute the closure is occasioned by an excess of muco-purulent secretion formed in the canal, thus preventing the normal entrance of air to the middle ear and causing marked deafness. The diagnosis of this condition is quickly made by forcing air into one nostril, the other being closed; while the patient pronounces the word "what" strongly, the hearing suddenly returns. If the hearing is not increased, or is but slightly increased, then the deafness is due to a thickened condition of the mucous membrane of the Eustachian tube. Frequently inflation is an injury in these cases because of there being no mucus to dislodge.

*An Abnormally Open Eustachian Tube* is not frequently met in this grade, and will be described in next older grade.

*Headaches* are common in this grade, especially with those going to school. Very frequently headaches are only symptoms of diseased nasal passages, or diseased ears, or abnormal condition of the eyes. The abnormal condition of the ears and eyes may be due to a nasal inflammation. If there is nasal inflammation present, it may very properly be assigned as the cause of any other affection of the patient at this age. I have seen this so often and for so many years that I feel sure that it will soon be conceded by the profession generally, as it is now by those who have made the subject a study.

The very close relation of the nose to the brain will account for some brain troubles following nasal inflammation. The very intimate connections of those two organs by blood-vessels indicate this. The blood supply to the upper two-thirds of the nasal cavities, to the sphenoid and ethmoid cells and to the superior and middle turbinates is received from within the brain cavity.

The anterior cerebral artery gives its first branch, the ophthalmic, to the eye; this sends two large branches to the nasal cavities and to the cells named. The first of these branches, the posterior ethmoidal artery, passes through the posterior ethmoidal foramen, supplies the posterior ethmoidal and the sphenoidal cells, and ascending, enters the cranium, gives off a meningeal branch which supplies the adjacent dura mater, then descends and again entering the nose through an aperture in the cribriform plate, is distributed to the superior turbinate processes. The anterior ethmoidal artery, also a branch of the ophthalmic, passes through the anterior ethmoidal foramen, supplies the anterior ethmoidal cells, and entering the cranium, divides into a meningeal branch, which supplies the adjacent dura mater, and a branch which descends and again enters the nose through an aperture in the cribriform plate, to be distributed to the middle turbinate processes.

It is thus seen that the anterior portions of the meninges are stitched, as it were, by these arteries to the upper two-thirds of the nasal cavities, giving an unexcelled opportunity for the transference of diseased action from the nasal passages to the brain. And experience abundantly proves that this frequently happens.

This nasal and brain circulation also explains how it is that the eyes are frequently affected as a sequence of nasal inflammation; it also explains how it is that school children are often unable to successfully study their lessons. They cannot retain in memory what they repeatedly read over, and the mind has a tendency to wander, much against their will, from their lessons to trifling matters.

This condition of the mind is frequently called "nervous prostration," and it is generally conceded that the pupil has been pursuing his studies too arduously, and a vacation is recommended. Of course a vacation will be beneficial, but it is evident the treatment of the primary trouble—the nasal inflammation—will be far more scientific, as well as far more effective.

No healthy young person can study too arduously, or too long. Nature's restorer, sleep, comes to the rescue, and on waking in the morning the student is as fresh as ever. I fully believe that the pathological condition of every one of such students compels the brain to resist a continuance of the studies, and that it is more or less dangerous to allow this condition to continue indefinitely.

*Mental Symptoms* are not sufficiently frequent in this grade to require an explanation of their pathological causes.

